# Sticking choices in timpani sight-reading performance

**DOI:** 10.3389/fpsyg.2023.1188773

**Published:** 2023-09-14

**Authors:** Benjamin Bacon, Stuart Jackson, Ian Marci, Fabrice Marandola, Marcelo M. Wanderley

**Affiliations:** ^1^Audio Communication Group (EN), Technische Universität Berlin (TU Berlin), Berlin, Germany; ^2^McGill University, Montreal, QC, Canada; ^3^Independent Researcher, San Rafael, CA, United States

**Keywords:** bimanual action, music performance, sight reading music, music notation, hand dominance, percussion, percussion performance

## Abstract

When sight-reading a score, a timpanist needs to decide in real-time which stick to use to play a specific note while interpreting the musical material. Our main point of inquiry seeks to understand which sticking patterns performers employ and how they are affected by rhythmic stability. This paper analyzes the bi-manual sequencing (i.e., sticking) patterns of 31 timpanists in a sight-reading task. We analyze their results compared to model sticking patterns common in percussion pedagogical literature. Results show that while hand dominance plays an essential role in an individual's sticking pattern, the stability of a rhythmic pattern may also dramatically influence the observed particular sticking strategies. In areas of rhythmic stability, performers largely adhered to one of two conventional sticking patterns in the literature (dominant hand lead & alternating). Where rhythmic patterns became more unstable, the performers separated into diverse sticking groups. Moreover, several performers demonstrated sticking patterns which were hybrids or an inverse of the model sticking patterns, without any impact on the success of their sight-reading abilities. Overall, no two individual performers demonstrated the same sticking pattern. In terms of percussion pedagogy, our findings suggest that performers may benefit from an awareness of the adaptability of model sticking strategies. Lastly, we make the case for further study of rhythmic stability and bi-manual sequencing by locating the difference between notational and aural complexity.

## 1. Introduction

Percussion performance primarily consists of players striking idiophones or membranophones with sticks, mallets, or hands to produce a sound. After the percussionist sets the instrument vibrating, they have little control over the sound. For this reason, the kinematics of the stroke defines the acoustic properties of each note (Stone, 1935; McCormick, 1983). Thus, for control of overall sound production in percussion, coordination between both hands plays a crucial role in developing the skills to perform well as a percussionist. In many ways, percussion performance is the artistic mastery of bi-manual coordination.

Timpani (also known as kettledrums) are one of the many instruments that percussionists need to master in terms of professional competencies. This type of drum consists of a large hemispheric bowl covered by a thin membrane that can be tuned and requires clear, deliberate and even gestures from both hands to achieve a desirable and consistent tone (Batigne, 1997): Inconsistent striking areas on the playing surface of the timpani, as well as minute changes in striking gestures, can produce variations in tone production (Chen et al., 2016).

Scores may often call for multiple drums (timpani) rather than a single one (timpano). When more than one drum is required, complex coordination and advanced sticking techniques are necessary to smoothly transition between instruments without disrupting sound quality. Such transitions, otherwise known as cross-overs, are focused on lateral movement across the body to preserve sticking choices while minimizing unintended strikes against the instrument or sticks themselves. Due to the long and resonant tone of a timpano, timpanists may often use their hands to dampen or mute the drum-head depending on the indicated duration of a given note. Lastly, concerning the size and pitch ordering of multiple timpani, there are two main methods; German and American ordering. In the American method, the pitch ordering is from left to right (low to high), in the same orientation as a piano. The German method is reversed, with the highest pitch on the left and the lowest on the right (high to low) (Montagu, 2002).

Given the complexity of the technique when performing across multiple timpani and the novelty of our study, we have limited our scope to observing performances on a single timpano. Our intention is to anchor our findings more closely to general percussion performance techniques.

## 2. Related works

While there have been many studies involving the percussion performance and sight-reading, previous works have not explicitly focused on sticking choice. Past research on percussion performance has primarily focused on the timing abilities of percussionists (Fujii et al., 2010), the kinematics of playing (Dahl, 2003; Fujii and Oda, 2009; Fujii et al., 2009; Fujisawa and Miura, 2010), or the effects of training on the sense of timing (Manning and Schutz, 2016).

Previous work directly related to this study involved an experiment with a sight-reading task which found kinematic and functional differences between the hands during percussion performance caused by handedness (Bacon et al., 2014). This study showed a clear correlation between rhythmic function and sticking choice. The players gravitated toward using the preferred hand for metrically important notes. The current study builds upon these findings with a larger pool of participating performers to explicitly test the effect of rhythmic complexity on sticking choice.

In the following subsections, we take a closer look at the surrounding literature informing our experimental focus.

### 2.1. Sight-reading

Sight-reading is often described as playing a piece of music that the player has never seen before, with the goal of performing all the aspects of the music as written, including correct pitches, rhythms, dynamics, and articulation markings. More specifically, sight-reading is a complex task involving fine motor skills in coordinating movement, active-memory recall when recognizing rhythmic structures, and the visual decoding of notation (Parncutt and McPherson, 2002). When sight-reading a musical score, the lack of preparation time allows an observer to witness a performer's skill level and natural performance tendencies. Performers also use sight-reading to reveal strengths and weaknesses in their playing technique and to prioritize practice time for future skill development.

It has been shown that complex sight-reading tasks require players to rely on their information-processing abilities more than their instrumental training (Kopiez and Lee, 2008). Furthermore, concerning sight-reading rhythm, a distinction can be made between an accurate rhythmic performance and what may be considered a precise clock-like internal pulse (Farley, 2014). When evaluating rhythmic content in a sight-reading exercise, barring any notable shifts or distortions in tempo, an accurate performance preserves the duration of a note in relation to its sequencing (Falle, 2011).

Current research in eye-hand coordination suggests that the distance between the gaze position of the eye in a musical score and the current position of the hands, otherwise known as the eye-hand span (EHS), can be used as an accurate evaluative method for sight-reading skill (Perra et al., 2021). In more experienced players, eye-gaze can scan further beyond their current playing position than their less experienced counterparts. Regarding notational density and tempo, score complexity plays a critical role in determining EHS distances. In terms of visual layout, spacing modifications between musical phrases in the notation of a score have been shown to increase musical legibility by demonstrating fewer sight-reading errors than unmodified notation (Stenberg and Cross, 2019).

Regarding performance expectations, there remains a strong contrast between a rehearsed, and sight-read performance. While flawless execution and creative expression remain the ultimate goals for both scenarios, the tolerance of mistakes is remarkably lower in the context of a rehearsed performance than in a sight-read one. While just a few misplayed notes could be viewed as detrimental in an orchestral solo amongst a sold-out crowd, those missed notes could be considered minor footnotes of an otherwise remarkably successful sight-reading demonstration. Moreover, while it is common in sight-reading that a single mistake may produce a string of errors, the ability to recover and conclude a given exercise confidently can also be viewed as a highly positive outcome when sight-reading. This does not minimize the stakes for common sight-reading scenarios. Sight-reading is commonly used in exams, auditions, and private lessons. Thus, while the expectations may differ concerning the ratio of correct/incorrect notes performed, the psychological pressure to perform well in the case of a sight-reading context may feel just as high as that of a high-profile performance.

### 2.2. Notation and rhythmic complexity

Western music notation can be seen as the intermediary between the composer and the performer. The notation communicates how a musical idea is to be performed. This can be done prescriptively in terms of what specific methods are to be used to recreate a desired sound (e.g., hit a piece of scrap metal with a hammer), or this can be done descriptively, where the notation represents the literal sound to be created (e.g., a G-sharp on the piano). Oftentimes, scores contain a mixture of the two (Kanno, 2007).

The wide variety of approaches when using Western notation speaks to the importance of standardization and the development of idiomatic forms of representation (Watson, 2006; Dimpker, 2013). At times, a piece of music may be notated in ways that, through a conflation of parameters, produces something overly complex from the performer's perspective. For example, a simple rhythm can be represented with uncommon time signatures, tuplets, note groupings, rests, and phrases. There are instances where the notation can be so dense that the music becomes impossible to play accurately (Duncan, 2010). Oftentimes, these pieces are re-notated by the performer so that the intended sounds are preserved while simplifying the notation (Talgam, 2019).

In terms of perception, the complexity of a rhythmic construct can be described in terms of its stability, where there is the expectation that tones or rhythms will repeat on a regular basis (Bigand, 1997). A sense of stability is achieved when listening to a regular periodic musical event. Although that sensation can be disrupted by introducing irregularly timed events, the general sense of stability can persist (Large, 2000).

### 2.3. Sticking and symmetry

When it comes to percussion performance practice, symmetrical gestures between the hands (both with and without sticks) are routinely emphasized to ensure an even sound when alternating them during a performance (Cook, 1997; Timbert and Rivalland, 2007). Performing with just one's preferred hand consistently is a non-practical solution, both economically for the player and musically. Thus, many players seek to equalize the performative capabilities between the hands in an attempt to achieve ambidexterity. Nevertheless, asymmetry in the body is naturally fundamental. It is known as laterality, which refers to the preferential use of one side of the body over the other for specific functions, such as hand preference for writing or throwing, or performing a percussive instrument (Corballis, 1983; Annett, 1996). Laterality's effect on manual behavior can otherwise be known as handedness, which has been shown to influence the performance behavior of a percussionist (Bacon, 2014). In skill-based bi-manual tasks, musicians have also been shown to exhibit less asymmetry between the left and right hands than non-musicians (Jäncke et al., 1997).

To counteract the natural behavioral asymmetry caused by handedness, a central tenet of percussion pedagogy prescribes that students spend extra time practicing to bring their non-dominant hand up to the skill level of their dominant hand, oftentimes suggesting that the student repeats exercises with the non-dominant hand three times as much as the dominant one (Delécluse, 1969; McClaren, 1994). To help foster parity during training, method books provide systematic stickings (i.e., the ordering pattern of the hands) for each exercise that balance the emphasis on the hands (Stone, 1935; Delécluse, 1969; McCormick, 1983; Goodman, 2000; Gworek, 2017). Given the complexity of assessing handedness, performance-based tasks are more reliable in assessing the balance of dominance between the hands (Kopiez et al., 2010). Thus, the study of sticking choices offers direct insight into how the natural forces of hand dominance and a performer's musical preferences interact.

When reading a piece of music in percussion performance, one generally rehearses to search for an optimal sticking that provides the most rhythmically accurate and expressively appropriate performance. Although an even-handed approach to sticking is considered an ideal outcome, when choosing a sticking order, a performer must consider the rhythmic complexity of the score, the combination of strokes that can best render the desired rhythmic phrasing, and their own physical limitations. In addition, professionals and students alike aim to develop an intuitive approach to sticking so that when they sight-read, they are most likely to automatically coordinate their hands to maximize their success in reading the notation correctly (Timbert and Rivalland, 2007; Commission nationale des programmes de lenseignement musical, 2016).

### 2.4. Model stickings

Due to the value placed on symmetry in percussion performance pedagogy, it would not be surprising for there to be little preference for a particular hand to play a certain type of note. However, the functional roles of the hands are often distinct (Stone, 1935; McClaren, 1994; Bacon et al., 2014). While the gesture is ideally symmetrical, the hands do not always perform equal functions when performing music (Dahl, 2003). With this in mind, previous research has shown that one's handedness, either left- or right-handed, has no inherent musical advantage (Kopiez et al., 2012).

Throughout the history of percussion, players learn that performance is often improved when relying on the dominant hand for metrically functional notes within a piece of music: the dominant hand acts as an anchor while the other hand “fills in” (McCormick, 1983). This playing paradigm has become known as “right-hand lead” for right-handed percussionists and left-hand lead for left-handed players (Stone, 1935; McClaren, 1994). The non-dominant hand performs better when automated (Peters, 1986), so percussionists maximize their rhythmic accuracy and sound quality by focusing attention on their dominant hand. For this reason, right/left-hand lead is the most popular sticking strategy for professional percussionists (McCormick, 1983).

Despite its prevalence, right/left-hand lead is not the only sticking strategy utilized by percussionists. Hand-to-hand or alternating sticking is also commonly used in conjunction with or instead of right/left-hand lead (McCormick, 1983). Instead of using the dominant hand to anchor, each hand is used equally. However, the preference for one hand is not entirely absent from this strategy since the dominant hand usually initiates each rhythmic section (Bacon, 2014).

**Figure 1:**
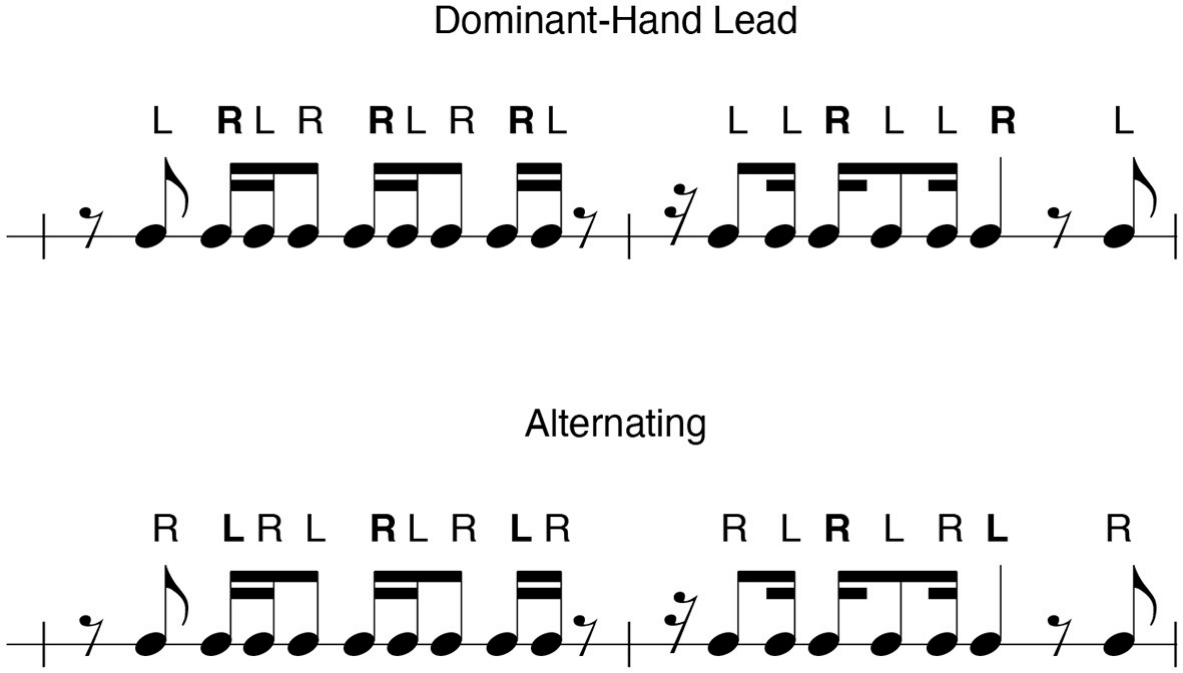
Examples of model stickings. In the dominant hand lead model, the right-hand performs metrically important beats, such as strong- and weak-beats (emboldened). In the alternating sticking, an alternating pattern is maintained throughout each measure regardless of the rhythmic content.

Given the importance of both sticking strategies in percussion performance methodology, dominant hand lead and alternating are two common sticking patterns that are often referenced in percussion method books and prescribed in pedagogical exercises (McClaren, 1994; Gworek, 2017). Exercises often provide multiple sticking patterns while advising that the student also practices the same routine while reversing their sticking to provide more training for the non-dominant hand (Gworek, 2017). In this way, they highlight as well as subvert the functional differences between the hands in order to improve performance in sight-reading and prepared literature. An example of differing sticking annotations for an identical rhythm is shown in Figure 1. Figure 1 shows one rhythmic excerpt with a right-hand or dominant hand lead sticking pattern annotated, and the same excerpt with an alternating sticking pattern annotated.

Despite the explicit references to the existence of preferred sticking in method books and teaching, there has yet to be an in-depth study to show if these sticking patterns are actually employed and how the employment of these model sticking patterns may change depending on the musical context. While there might not be a clear consensus on the best-sticking choices in timpani performance, there certainly are preferences toward these two model sticking patterns in the pedagogical literature. While professionals and students may use these sticking choices in practical performance situations, other factors, such as unidiomatic notation or more complex rhythmic structures, may disrupt this trend. Furthermore, players may sometimes use these model sticking patterns for some sections of a score and not others. In our study, we aim to provide explanations for how sticking patterns change with the musical context, particularly with sight-reading.

## 3. Research questions and hypotheses

We explore sticking patterns when sight-reading while performing on a timpano with the following research questions:

- RQ1: Do players mostly use one of the two model stickings discussed in the literature, or are other models commonly used?
- RQ2: How does rhythmic complexity affect sticking choices?

Based on these questions, we hypothesize that:

- H1) Sticking patterns tend to gravitate toward one of these two models, especially when encountering more typical and simple rhythmic passages, and stray from them as the rhythms become more complex.
- H2) Rhythmic complexity will affect sticking choice, with sticking patterns becoming more varied as rhythmic complexity increases.

We seek to differentiate musical context's effects on sticking patterns by dividing measures into those with stable or unstable rhythms. We predict that the rhythmically stable measures will yield fewer sticking patterns among the participants, while unstable rhythms will yield the greatest number of strategies.

## 4. Methodology

The experimental methodology employed in this study aimed to capture participants' natural sticking strategies during sight-reading. The study adopted a between-subject cross-sectional design, with individual distances from model stickings (Hamming Distances) serving as outcome measures, with a paired Wilcoxon Rank test used as an assessment of their statistical significance. The sight-reading score was designed as a traditional rhythmic étude, featuring sectional developments introducing new material. Its legibility and comparability to typical snare-drum or timpani études was confirmed by co-author F. Marandola, an Associate Professor of Percussion at McGill University. Following the sight-reading session, the authors transcribed individual sticking patterns and organized them in a spreadsheet for analysis and comparison with our model stickings and other participants.

### 4.1. Score design

**Figure 2:**
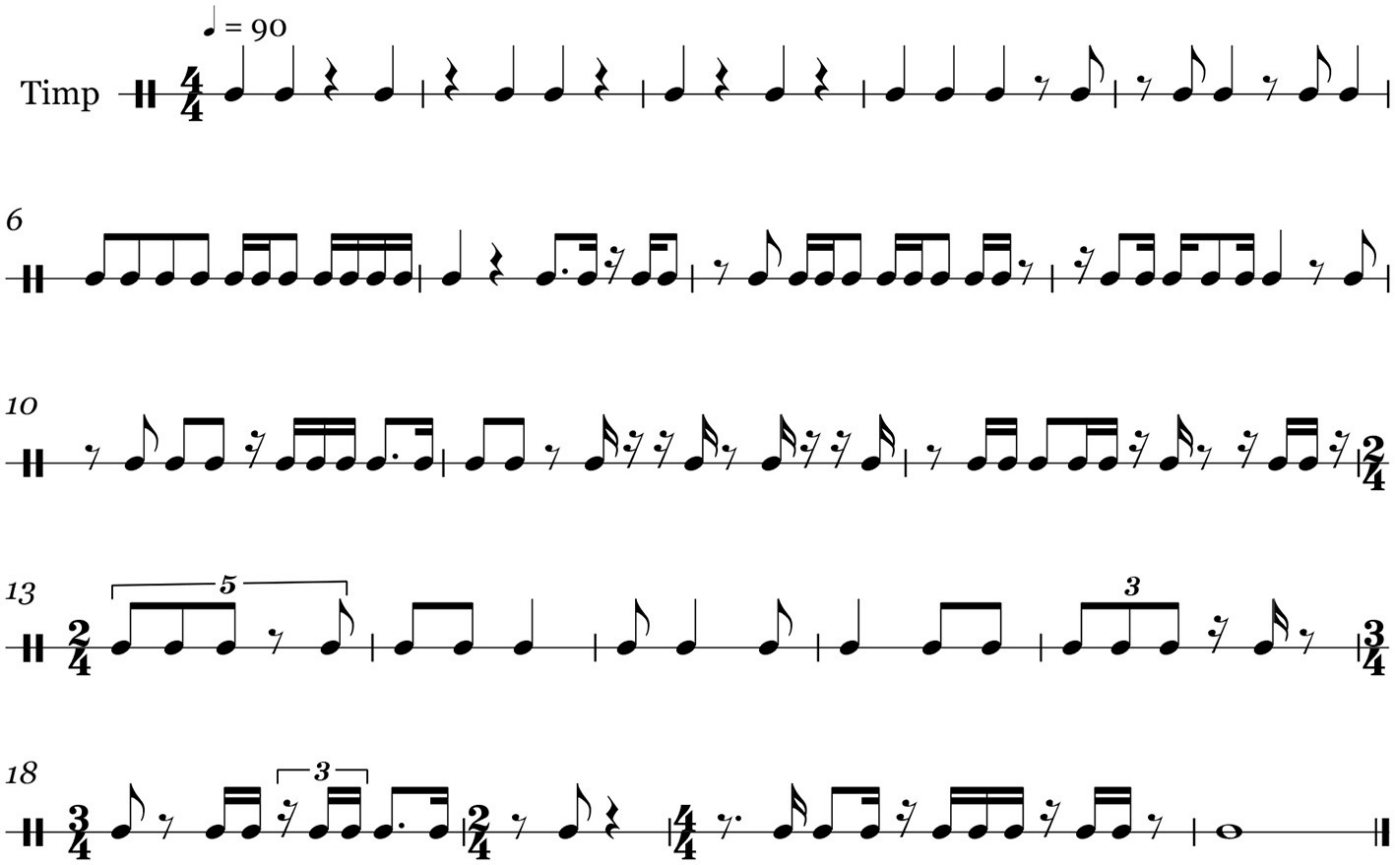
The entire sight-reading score used in this study, as seen by the participants.

Participants were given a score composed specifically for this exercise, shown in Figure 2. It contains rhythms representative of standard snare-drum and timpani repertoire and gradually increases in complexity (Bacon et al., 2014). Snare-drum literature was referred to in the design of this score due to the fact that, while the experiment was performed on the timpani, it was desired the performer operate comfortably in the context of a single drum, rather than considering more complex multi-drum environments. In addition, the score was written using the snare-drum standard template in the Sibelius 6 notation software. Lastly, the score was composed such that approximately equal numbers of notes are on the beat or syncopated, ensuring that direct comparisons could be made regarding hand choice for each note type.

The score's design was of particular importance, as it was imperative to challenge the performers ability to read while also introducing them to material which would not immediately produce a large string of errors, thus rendering their performances less useful in the context of our study. In addition, it was essential to consider if or how the score could introduce a bias toward either the left or right hand. Given the authors expertise and familiarity with percussion literature, we determined that the simple rhythm at the beginning of the score allows the performer to begin with either hand with minimal pressure or attention needed to be performed correctly. Furthermore, the score was clear of any ornamental notation, such as dynamics, articulations, rolls, or tempo changes, which would disrupt or discourage a simple alternating sticking pattern.

#### 4.1.1. Structure

The overall structure of the score can be broken down into nine sections of increasing and decreasing complexity mediated by syncopation and irregular rhythms. Syncopation can be generally described as rhythmic cues which fall outside or contradict an inferred musical pattern which has already been established (Lerdahl and Jackendoff, 1996). In the scope of our research, we define syncopation as notes which fall just after or before a beat or strong beat (i.e., an important demarcation of the time signature) but can still be located on a grid of sixteenth-note subdivisions. We also define irregular rhythms as another form of syncopation in our study as tuplets that do not fit into a grid of sixteenth-note subdivisions. Outside the scope of the audible quality of a given rhythm, the score also contains a strong example of unidiomatic notation in measure 11. The visual spacing of the notes and the excess use of sixteenth-note rests obfuscates where the actual metrical markers which govern the measure reside.

**Figure 3:**
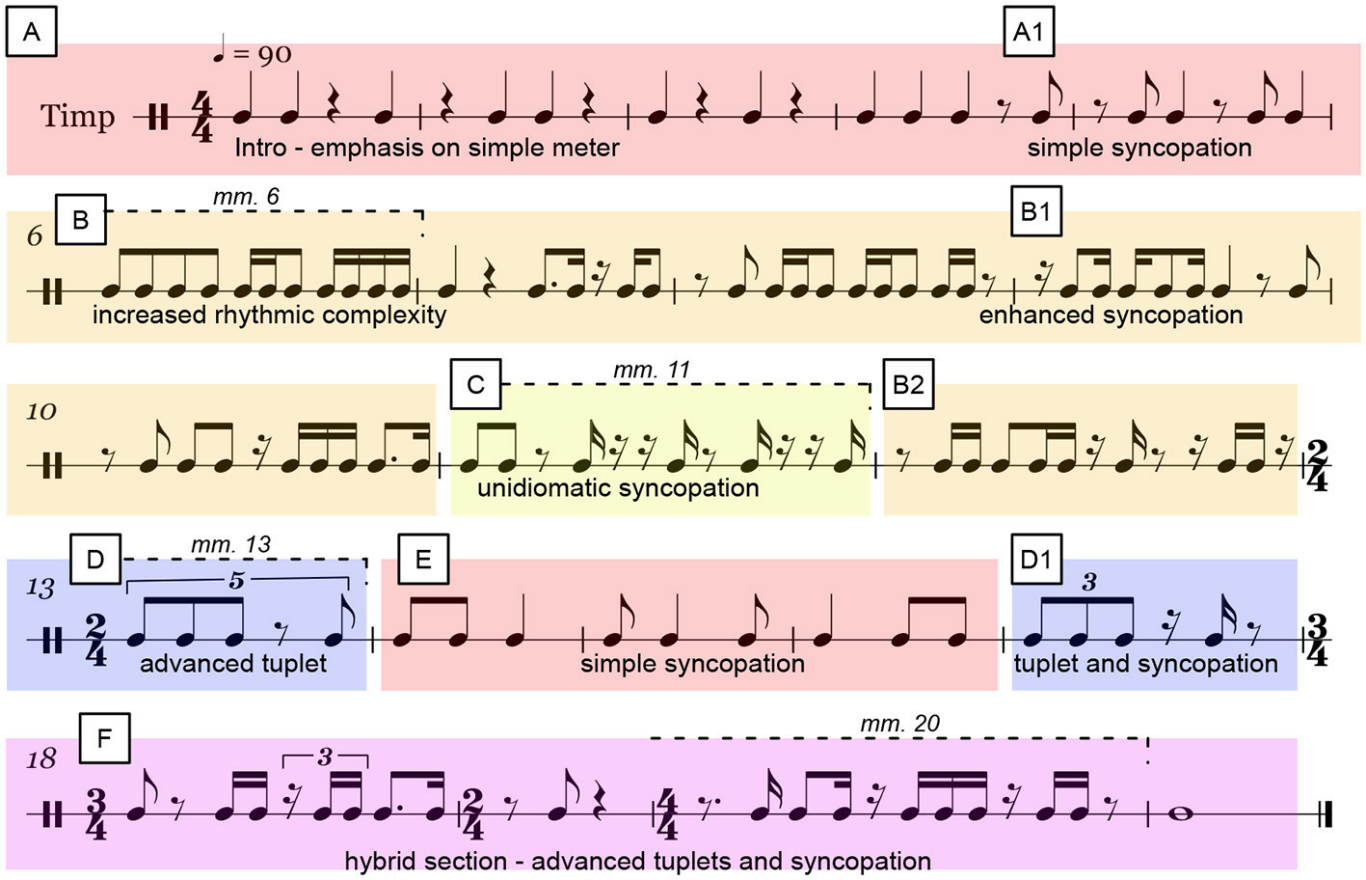
The various regions of rhythmic complexity can be seen. Color coding indicates each section in relation to musical complexity. Red **(A, A1)** relates to straightforward rhythms with minimal syncopation, followed by orange **(B, B1, B2)**, indicating further use of 16th-note subdivisions and more complex syncopation. Yellow **(C)** corresponds to complex syncopation represented by unidiomatic notation due to the groupings of 16th-note rests. Green **(E)** represents a reprieve to simple meter and syncopated patterns flanked by indigo **(D, D1)** which contains polymetric tuplets, and violet **(F)**, which contains hybrid elements of advanced tuplet use and heavily syncopated rhythms. Stable mm. 6, 20, and unstable mm. 11, 13 are marked by dashed lines.

Beginning with larger and simpler beat subdivisions, the score moves toward simple syncopation patterns and onto smaller beat subdivisions, complex syncopation patterns, and polymetric tuplets with periods of simple segments in between to provide a respite from the more difficult passages. The polymetric tuplets imply the existence of two overlapping metric structures; that of the tuplet (e.g., placing 3 or 5 beats in the space of 4) and that of the common notational grid. Further changes to the time signature itself consisting of 2/4, 3/4, and 4/4 time were also employed so that the performers would be challenged not only in their ability to keep accurate internal timing within a particular beat framework but also in their ability to adapt to new global timing structures in general. The insertion of simple segments in between more complex ones was done so that the performers could recover physically and mentally reorient themselves after periods of sustained musical complexity. The annotated score is shown in Figure 3.

**Figure 4:**
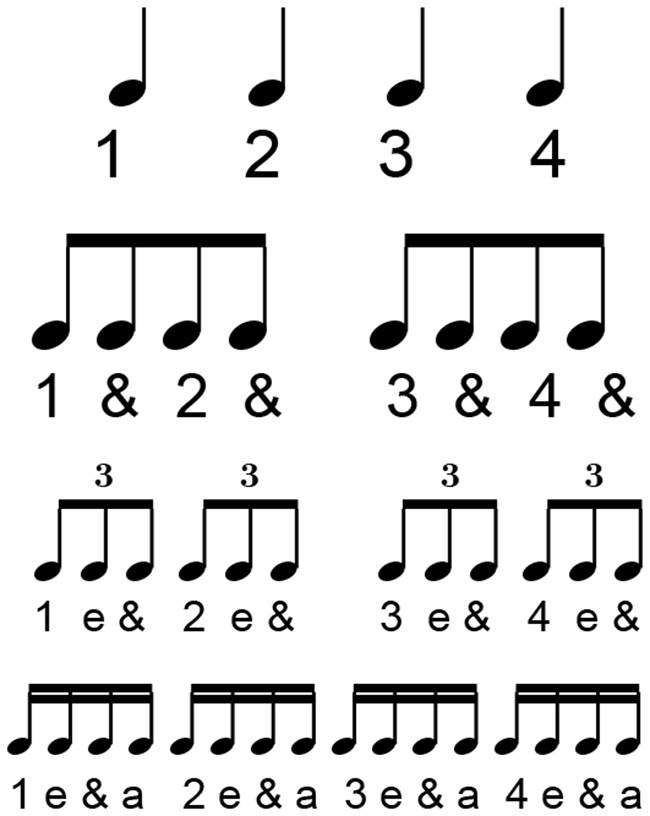
A measure containing four quarter notes subdivided into eighth notes and sixteenth notes. Notes labeled with a 1 are strong beats. All numbers are beats. “&” indicates an eighth note syncopation while “e” and “a” indicate sixteenth note syncopations with the exception of triplets.

Each beat in the sight-reading score can be evaluated using five different timing functions; strong beats, weak beats, eighth notes, sixteenth notes, and tuplets. Strong-beat and weak-beat notes consist of the primary beat values, often referred to as quarter-notes. In a 4/4 measure, these notes refer to each of the four main beats. In a typical subdivision of the beat in 4 sixteenth notes, the strong beat consists of beats one and three, while the weak beats refer to beats two and four; the eighth-note beats fall in between the strong- and weak-beat quarter-notes; the sixteenth-note values refer to notes which fall between the eighth notes, as seen in Figure 4; lastly, the tuplets in this exercise refer to irregular beat structures which cannot be evenly mapped to the grid structure of sixteenth notes. For example, the quintuplet (i.e., five notes under a bracket) found in measure 13, the sight-reading score places five eighth notes in the space of four.

In our study, we distinguish between the type of notation used to represent a specific beat and the beat function previously described, as we were not evaluating the sustained note values in the sight-reading score. In many cases, a strong beat can be represented by notation which does not indicate specifically where that beat takes place, as this refers to the length of time a note should be held. This is how we have defined the unidiomatic notation segments in the sight-reading score, which account for greater rhythmic complexity. For example, in measure 11, rests were split and grouped such as to produce an ambiguous sense of placement within the notational grid. This was done to direct the performer's attention toward maintaining their sense of internal timing, which in turn minimizes focus on expressive gestures and conscious sticking choices. In addition, standard notation formatting can be recognized by the performer and performed automatically through memorization. For functional purposes in this percussive context, we are only interested in sticking choices in relation to note-onset times.

#### 4.1.2. Rhythmic stability

Figure 4 shows how all the beats in a measure can be subdivided from quarter notes to sixteenth notes. Looking at the bottom line of this rhythmic tree, the sixteenth notes can be divided into their various parts with the designation 1 e & a, with the first number referring to the strong beat or beat in the measure, and “e & a” referencing the further sixteenth note subdivisions of that beat.

For our study, we define stable rhythms as patterns that fit into the 16th-note subdivisions in a given measure, with very little or no syncopation. Unstable rhythms use irregular patterns, syncopation, and unidiomatic notation, including rest groupings which may obscure clear metrical markings. Unstable rhythms are generated through a combination of high syncopation levels and unidiomatic notation representation.

Our study identifies musical passages as either stable or unstable rhythms to test the effects of rhythmic complexity on sticking choices.

### 4.2. Choice of stable and unstable rhythms

The overall structure of the sight-reading task consisted of several sections, which gradually increased in levels of rhythmic complexity. Although the score contains many interesting examples of rhythmic content, we have selected two stable and two unstable regions for our analysis from the larger sections B, C, D and F (as seen in Figure 3) to examine our second hypothesis. These measures are of particular importance in that they represent the introduction of unique rhythmic material.

#### 4.2.1. Stable rhythm 1–measure 6

Measure 6 is the first measure of Section B and introduces sixteenth-notes as well as the first elements of more advanced syncopation. Nevertheless, it represents a very typical rhythmic pattern that most percussionists will have seen and played many times before. Moreover, it contains no irregular rhythms which create tension with a sixteenth-note grid (i.e., tuplets).

**Figure 5:**
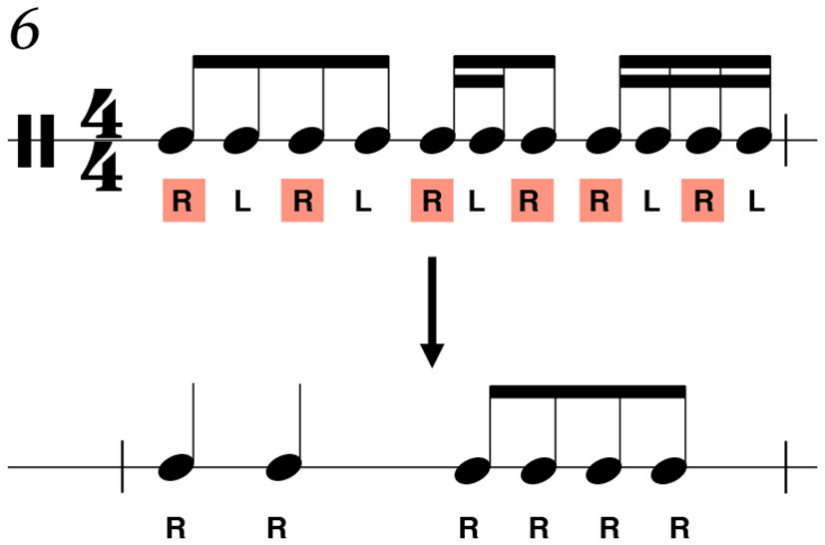
Measure 6 shows the part of the rhythm only played by the dominant hand. The highlighted R's are re-written as a reduction in the bottom half, demonstrating the larger beat functions.

Despite increased rhythmic complexity compared to the previous measures of the score, this section should produce a predictable sticking pattern due to there being no presence of rests, forcing more automated sticking choices onto the performer. With the dominant hand lead in particular, this measure invites this strategy more than others since the dominant hand can maintain a steady pattern. In contrast, the non-dominant hand fills in the sixteenth note subdivisions. This type of approach can be more clearly seen in Figure 5.

This predictability depends on the musical context and the fact that the preferred hand is better at producing consistent rhythm and that the non-preferred hand is better when automated (Peters, 1986). In measure 6, the larger subdivisions of quarter and eighth notes can be maintained steadily with the dominant hand. Because the music being performed is unrehearsed, the participants in the experiment will focus their attention on the oncoming notes, requiring the hands to respond automatically.

#### 4.2.2. Stable rhythm 2–measure 20

Measure 20 is another example of stable rhythms which are more complex than the ones shown in measure 6. This measure is important as it presents a return to the original time-signature after several measures of changes in meter (from 2/4, 3/4 back to 4/4 time). Though syncopation is present, particularly on the first beat, all the notes fit into the sixteenth note grid and are played on every strong and weak beat. These constitute the main grid markers of the time signature.

#### 4.2.3. Unstable rhythm 1–measure 11

Measure 11 (section C) is unique, as it was specifically composed to contain the unidiomatic representation of syncopation, where the rhythms were designed to throw-off the performer. The overabundance of sixteenth-note rests were placed to deliberately make it unclear to the performer where the strong-beat is, making it more difficult to read.

#### 4.2.4. Unstable rhythm 2–measure 13

Measure 13 offers the first example of a rhythm that we consider to be unstable due to the presence of a tuplet. A single quintuplet which does not fit the grid of quarter-note, eighth-, or sixteenth-note subdivisions makes the automation that would occur with a dominant hand lead strategy difficult and puts this in the category of an unstable rhythm. Here we introduced a silence within the tuplet to increase the challenge for the performers.

### 4.3. Participants

A total of 31 participants were selected for the sight-reading task with at least one year of university-level training in percussion performance. The average age of the participants was 25.09 years of age with a standard deviation of 6.83 years. This group of participants, with many years of performance experience, can be considered quite advanced with an average of 11.81 years playing experience. Of the 31 participants, five self-identified as left-handed (P6, P7, P8, P10, and P30), and the other 26 identified as right-handed, further referred to as P1-P31. Regarding grip style of the timpani mallets, all participants employed French-style grip except for 3 (P2, P26, and P30) who employed German grip, and 2 (P4 and P13) who employed a hybrid French/American grip. All participants resided in the greater metropolitan area of Montreal, QC, Canada and were recruited over the course of three experimental periods. In addition, in each phase, all participants performed the sight-reading on a single drum with their own timpani mallets of medium-firm hardness. The first round of data included participants P1-P4 (October–November 2013) seen in Bacon (2014), the second included participants P5–P15 (November 2015–February 2016) seen in Marci (2018), and the third and most recent data phase included participants P16-P31 (June 2021–April 2022).

### 4.4. Experiment procedure

Participants were asked to get ready behind a single drum with their mallets in preparation for performing the sight-reading task. All experimentation phases were conducted in accordance with the REB-II ethics protocol from the McGill University Ethics Committee.

The participants were instructed to employ their preferred technical grip and to place less focus on rigorous timekeeping established by the metronome, which was set to 90 beats per minute. Performers were instructed to focus on the onset times of the rhythms in the score, with the ultimate goal being that each participant performs relaxed and comfortably.

The experiment began with the score presented in Figure 2 sitting on a music stand face down so the performer could not see the musical content of the score. When the performer was ready to begin, a proctor pressed play on a video camera, and the proctor turned over the score. Immediately after this, the metronome was used to count in the performers and was switched off at the beginning of the 4th measure. To analyze the performances, the video footage of each performer was extensively reviewed to manually compile statistics on left- and right-hand strokes, identify errors in the performer's play, and search for other unforeseen effects of handedness.

The timpano they performed on was based on the availability at the time of the experiment, which took place either at a research lab or a percussion studio. However, since the sight-reading score contained no pitch information, the fact that participants performed on different-sized timpani did not notably affect their sticking choices since they were only performing rhythms and not engaging with the drum's ability to tune. This is further bolstered by the fact that percussionists are accustomed to practicing and performing on different models as students and professionals. If the notation had required specific tuning changes, the drum size would take on much more significance, as this fact limits the range of available pitches. Tuning changes on the timpani also elicit a broader set of gestures from the performer (such as leaning closely to the drum to better sense the accuracy of the new pitch's intonation).

Following the experiment, the participants were invited to provide brief evaluations of their playing performance quality throughout the experimental process. This step was added to ensure that recorded results were the product of each performer's natural playing style.

### 4.5. Data analysis

Participants' sticking patterns were annotated from the videos of their performances into the score. Transcriptions of sticking choices were done by hand by the authors.

By following along with the score and watching each participant's recorded video, sticking patterns were written in the score to correspond to which hand played which note so that by the end, we had separate scores for each participant with their individual transcribed sticking choices annotated in the score.

Notes performed by the dominant hand were labeled pink, while notes performed by the non-dominant hand were labeled gray. This enabled us to have a visual overview of all the participants sticking choices for the entire score and derive statistical information regarding sticking choices concerning the beginnings of defined regions, beat type, number of sticking changes, and error rates.

Following that, certain regions of the score were extracted based on the stability of stable and unstable rhythms. We selected two stable regions (measures 6 and 20) and two unstable regions (measures 11 and 13). Once a region was chosen, we calculated error rates for that region and then reorganized the spreadsheet based on identical sticking patterns so that it would be clear to ascertain how many participants employed a given sticking pattern or how varied the sticking patterns were in a given region.

Data was analyzed by comparing sticking patterns with model sticking patterns using a threshold for errors and error classification. Score analysis was done using a classification of note and beat types and how they typically function musically. These issues are discussed in the following sections.

In this study, we used both dominant hand lead and alternating sticking pattern models as found in the literature. The model patterns serve as default sticking patterns to measure predictability in sticking choice among the participants.

#### 4.5.1. Error classification

In identifying errors, two types of classification emerged: mistimed strikes and ghosted strikes. Mistimed strikes are defined in this study as attempted notes in relation to note-onset which were clearly outside the rhythmic time frame indicated by the notation. In the rare case where multiple mistimed strikes occur for a specific beat, these instances were recorded as a single error. Ghosted strikes are omitted notes from the score, either as initiated strikes but restrained from making contact with the timpano membrane or entirely skipped altogether.

Because a certain number of errors are to be expected when performers are engaged in a sight-reading task, we have placed a threshold on the number of mistakes we accept for each participant when discussing the results of certain data sections. For this work, we chose not to consider any participants that missed more than 20% of the notes in a given section of the score, either making a timing-based error in rhythmic execution (mistimed strikes) or omitting a note (ghosted strikes). This threshold was chosen based on the same principles applied to a student engaged in a sight-reading exam, where a certain number of errors are allowed, but if the errors reach a certain threshold, they would fail the exam. Full details on the number of removed participants per section of the score can be seen in **Table 2**.

Lastly, due to the absence in the notation of any articulation markings and sustained rolls on the drum-head, slight variations of the drum playing surface were not considered to have any effect on the participants ability to conduct the task whatsoever and were not considered errors.

#### 4.5.2. Beat classification

**Figure 6:**
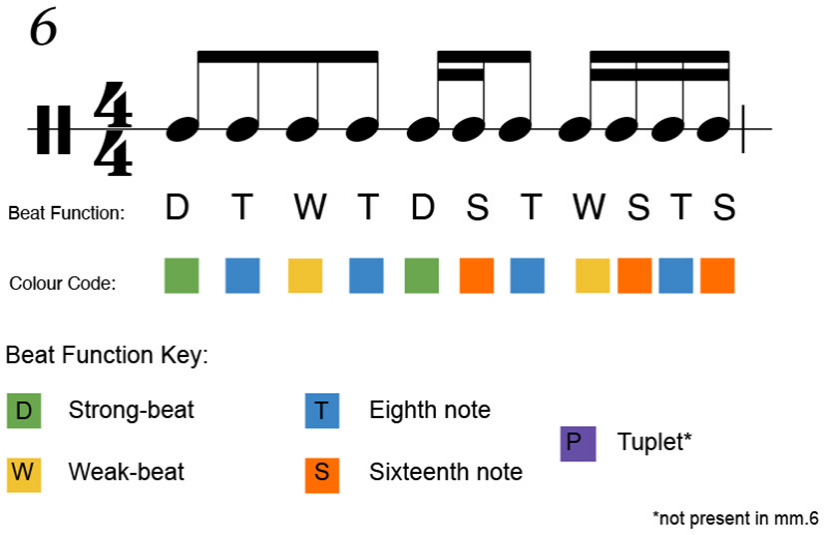
Labeled notes of the sixth measure of the score. The measure contains two strong beats, two weak beats, four eighth-note syncopations, and three sixteenth-note syncopations. Color coding is seen in sticking charts. No tuplets appear in measure 6.

As shown in the score structure section of the paper, each note of the score was classified according to its metrical function. Strong beats, weak beats, eighth notes, sixteenth notes, and tuplets were annotated in the score as aids for analysis of the sticking patterns chosen by the performers. This provided a hierarchical map that enabled us to correlate hand preference to beat type. An example from the score can be seen in Figure 6. Table 1 shows the prevalence of different beats and rhythmic elements in the entire score.

**Table 1:** Distribution of rhythmic elements in the entire score.

**Element**	**% of Score**	**Function**
Strong-beat	20.39	Demarcates measures and half-measures
Weak-beat	20.39	Basic beat marker
Eighth note	25.24	Introduces syncopation
Sixteenth note	27.18	Enhances metered complexity
Tuplet	6.79	Obscures metering

The performance videos were annotated to determine which hand each percussionist used to play each note. The percentages of dominant and non-dominant strokes were computed for each of the five metrical groups.

## 5. Results

Percussionists strongly preferred to use their dominant hand for strong beats with an average 69.99% use rate for all instances in the score. In both strategies, the dominant hand starts the beginning of phrases. Despite this trend, no two players used the same sticking pattern throughout the score. Nevertheless, it is possible to find several sections where the same sticking pattern is repeated among participants. The more homogeneous sticking patterns were observed to reproduce the two model sticking strategies we referenced earlier in the paper, with the dominant hand lead strategy yielding more sticking similarities with the participants across the entire score. When model sticking patterns were not strictly adhered to, other strategies emerged either as hybrids between alternating or dominant hand lead or strategies that did not resemble the model strategies.

Furthermore, we found that musical sections composed specifically to contain unstable rhythms led to more varied sticking patterns, confirming our second hypothesis. More rhythmically stable passages generally yielded a higher percentage of model sticking strategy usage among participants and more homogeneous sticking patterns. In contrast, rhythmically unstable passages resulted in much less utilization of model sticking patterns and a greater variety of sticking strategies overall.

### 5.1. Interpreting errors

In general, all the participants performed the score well: 14 participants executed an error-free performance, while seven others made fewer than three errors. The total error rate across all participants was 3.75%. Throughout performing the score in its entirety, only Participant 30 (P30) crossed the 20% for the entire score at 22.33%. When analyzing specific regions of the score, participants that locally made more than 20% of errors were removed. This was necessary to identify the sticking patterns being used, as a high number of errors would lead to a lack of clarity in data analysis. Table 2 shows a full readout of omitted participants per section.

**Table 2:** Omissions from sectional statistics due to crossing the 20% error threshold are seen above. Only performer P30 exhibited an error rate above 20% for the entire study at 22.33%.

**Section**	**% of score**	**Participant(s) P**	**Note range**
A	9.71		1–10
A1	4.85		11–15
B	24.27	13	16–40
B1	14.56	12, 13, 15, 30	41–55
C	5.83	30	56–61
B2	7.77	10, 19, 21, 24, 27, 30	62–69
D	3.89	19, 27, 30	70–73
E	8.73	19, 21, 24, 27, 30	74–82
D1	3.89		83–86
F	16.50	7, 19, 30	87–103
mm. 6	10.67		16–26
mm. 11	5.82	30	56–61
mm. 13	3.88	19, 28, 30	70–73
mm. 20	7.76	17, 19, 23, 30	95–102
Full Score		30	1–103

In the following sections, we will discuss the stroke distribution at the start of each section, compare similarities in sticking for the entire score between the participants and the model stickings, then detail the striking choices for two stable regions (measures 6 and 20) and two unstable regions (measures 11 and 13).

### 5.2. Similarity to model stickings

Across all participants, we have made a direct comparison between the model stickings and the observed sticking choices of the participants in our study. Given the bi-manual nature of percussion performance, sticking orderings could be seen as a binary string consisting of dominant and non-dominant hand use in the case of our study. Therefore, in a direct stroke-for-stroke comparison, the maximum number of different sticking choices between a participant and a given model sticking is 103; the total number of notes in the sight-reading score, otherwise known as the Hamming Distance (Toussaint et al., 2004). The average distance from the model stickings across all participants and the standard deviation can be seen here in Table 3.

**Table 3:** Similarities of model stickings to performed stickings across all performers including results from a paired Wilcoxon Rank test. The total possible strokes in the score is 103.

**Model sticking**	**Hamm Dist**	**Hamm % of Score**	**SD of Hamm**	**W statistic**	***p* value**
Alternating lead	57.00	55.33	9.96	40.5	< 0.001
Dom Hand lead	36.57	33.50	17.72		

Participants showed greater stroke-for-stroke similarity with the dominant hand lead model sticking but with considerable variation between participants; the standard deviation of similar stickings between the participants was 9.96 strokes for the alternating model sticking and 17.72 for the dominant hand lead. The altefrnating hand pattern measured an average of 57 differences with the participants or 55.33% of the 103 possible notes in the score. In comparison, the dominant hand lead stood at 36.57 sticking differences or 34.5% of the total possible notes. A paired Wilcoxon Rank test demonstrated that this difference is highly significant (W = 40.5, *p* < 0.001).

#### 5.2.1. Similarity in stable and unstable measures

In the example measures of stable and unstable rhythms shown in Table 4, we again find that the participants exhibit a closer similarity to the dominant hand lead pattern overall, with the closest stroke-for-stroke similarities found in measure 13. In this case, the average hamming distance between the dominant hand lead sticking and the performers was 3 out of 11 strokes or 27.27% measure. The stable rhythmic quality of measure 20 contained the smallest difference between alternating and dominant hand lead model stickings at 4.07 and 3.96 strokes, respectively. These hamming distances correspond to 50.87% stroke difference for the alternating lead and 49.50% for the dominant hand lead. The lack of significance was also evident with regards to the Wilcoxon Rank test results (W = 32.5, *p* >0.5).

**Table 4:** Stroke similarity (i.e., hamming distance) comparisons of stable and unstable measures with the model stickings.

**Quality**	**Measure**	**Strokes**	**Model**	**HD**	**HD %**	**SD**	**W statistic**	***p* value**
Stable	6	11	Alt	7.52	68.36	3.30	80.0	< 0.001
			Dom lead	3.00	27.27	3.69		
	20	8	Alt	4.07	50.87	2.20	32.5	>0.5
			Dom lead	3.96	49.50	2.03		
Unstable	11	6	Alt	3.83	63.83	1.76	126.0	0.027
			Dom lead	2.47	41.16	1.80		
	13	4	Alt	3.07	76.75	1.12	31.0	< 0.001
			Dom lead	1.07	26.75	1.25		

Hamming distances are displayed in stroke values, and as a percentage of notes in the given measure. In this table, the alpha level for significance was reduced to 0.0125 to correct for multiple testing.

Overall, the four measures analyzed here represent a wide range of note values, from the largest measure note count in measure 6 (11 notes), to one of the smallest in measure 13 (4 notes). Despite difference in rhythmic stability and note count, measure 6 and measure 13 contained a similar balance in hamming distances with the two model stickings. In fact, both measures demonstrated highly significant differences between alternating- and dominant hand *p* values visible in Table 4.

### 5.3. Muting

Although participants were instructed to perform only note-onset times (i.e., not to influence note duration), muting was observed in 22.58% or 7 out of the 31 participants; P17, P19, P21, P23, P26, P27, and P30. In each case, the instance of muting was subtle and not consistently implemented throughout the reading of the score. Regarding sequences of single-hand use, the performers who exhibited instances of muting demonstrated an average longest dominant hand sequence of 5.71 strokes (out of a possible 103), as opposed to the non-muting average of 4.20 strokes. Muting performers had a slightly higher than average dominant hand performance rate at 54.91% as opposed to the non-muting average of 53.88%. In addition, muting performers demonstrated an average error rate of 9.01% as opposed to the average of 2.22% for non-muting players. Lastly, of the seven players who exhibited muting, only one was a self-identified left-handed player, which is consistent with the natural distribution of left-handed persons in the general population.

### 5.4. Initial stroke distribution

At the start of each section, it appears that a given note beat function has an influence which hand is used to begin the segment. The first strong beat is 90% dominant hand initiated, while the following segment first note is an eighth note, pushing the dominant hand starting percentage to 50%. This trend continued with the beginnings of other sections. For example, at the beginning of section 3 (increased rhythmic complexity), the first note is 84% dominant hand initiated.

Table 5 shows the major sections of the score in order of appearance. The type of beat each region begins with and the percentage of players that used their dominant hand to play that beat are shown.

**Table 5:** This table displays the dominant hand use on the first note of each section of the score.

**Section**	**Beat function**	**Dom hand use %**	**% diff Alt**	**% diff DHL**
A	Strong-beat	90.32	9.68	9.68
A1	Eighth note	48.39	51.61	48.39
B	Strong-beat	83.87	83.87	16.13
B1	Sixteenth note	41.94	58.06	48.39
C	Strong-beat	67.74	70.97	32.26
B2	Eighth note	77.42	77.42	22.58
D	Strong-beat	93.55	93.55	6.45
E	Strong-beat	70.97	80.65	29.03
D1	Strong-beat	74.19	25.81	25.81
F	Strong-beat	67.74	32.26	32.26

The beat function column indicates the kind of rhythmic function present at the start of each section, followed by the use rate of the dominant hand across all performers for that given note (Dom Hand Use %). The last two columns written as “% diff Alt” and “% diff DHL” reference the percentage of performers who did not agree with the model stickings. P30 has been included in this case due to a correct stroke occurring on each section's initial beats.

These results indicate a preference for the dominant hand when playing strong beats. Note that with the sixteenth-note subdivision within the Enhanced Syncopation section, the preference for the dominant hand drops to 41.94%, the lowest percentage for any initial note in a given section.

### 5.5. Stable rhythm 1–measure 6

Measure 6 is an example of a stable rhythm pattern that is common enough to yield a predictable sticking strategy from most percussionists in a sight-reading task and is relatively easy to perform. In this measure, shown in Figure 6, we found that a dominant hand lead sticking pattern was the most common strategy used, followed by an alternating sticking strategy. This aligned with our first hypothesis, as we expected the two model sticking strategies to be the most commonly used by participants for this measure.

All of the participants in this section were included for consideration, as the error rates never reached or exceeded the 20 percent threshold. For measure six specifically, 16 out of 31 Participants employed a dominant hand lead sticking strategy, 9 employed an alternating sticking strategy starting with the dominant hand, and 3 employed an alternating sticking strategy starting with the non-dominant hand. Interestingly, P2 and P6 used a non-dominant hand lead sticking strategy. P8 used a different strategy altogether.

**Figure 7:**
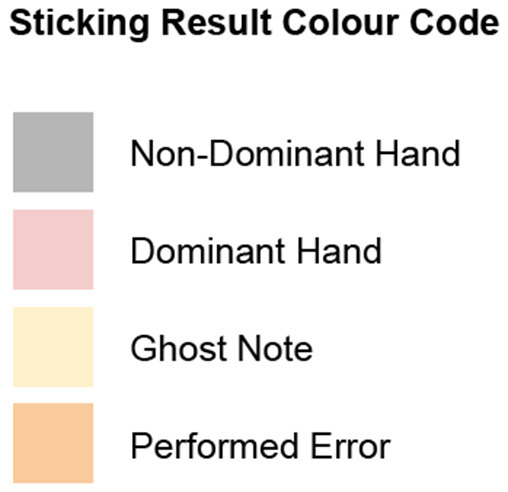
Color codes used for notating hand choices and errors.

The following charts group together the participants who used identical sticking patterns. Pink squares represent the dominant hand (lighter gray when printing in B&W), and gray represents the non-dominant hand. Orange squares represent errors, and yellow represent ghost notes (notes not played). Numbers under the column labeled “note” correspond to the note number, as seen in the measure reproduced below. A legend describing the color coding of the sticking charts can be seen in Figure 7.

**Figure 8:**
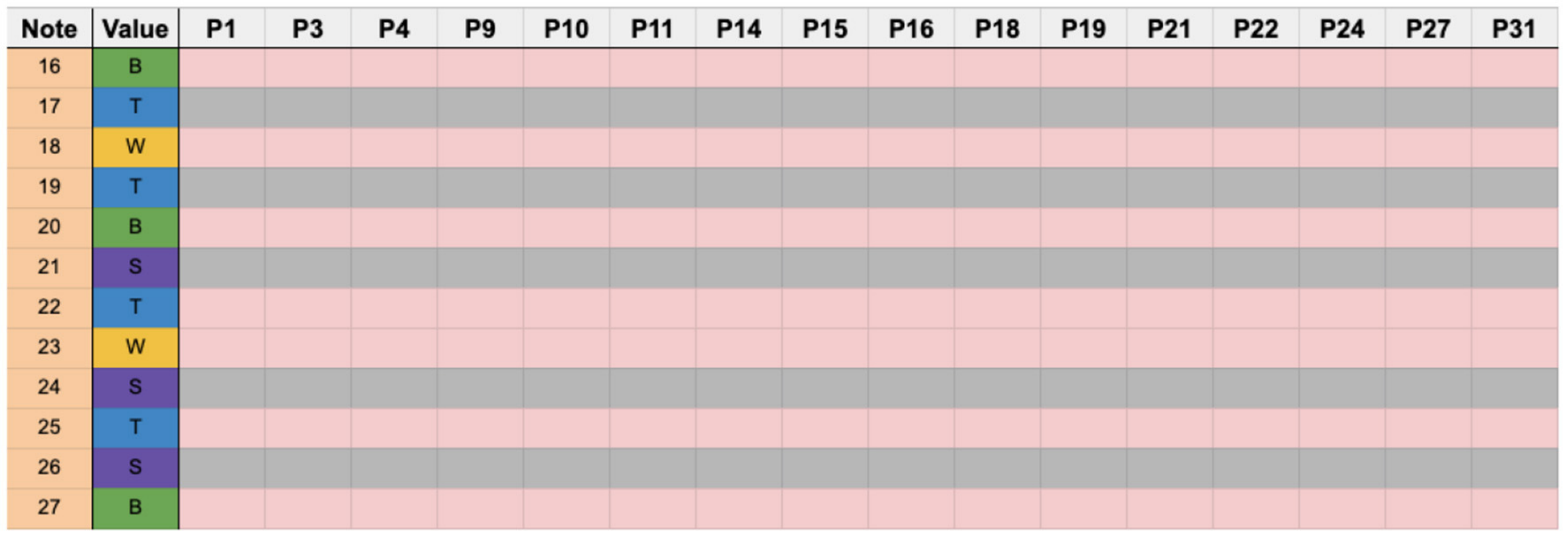
Chart showing the participants that used a dominant hand lead strategy for measure 6.

**Figure 9:**
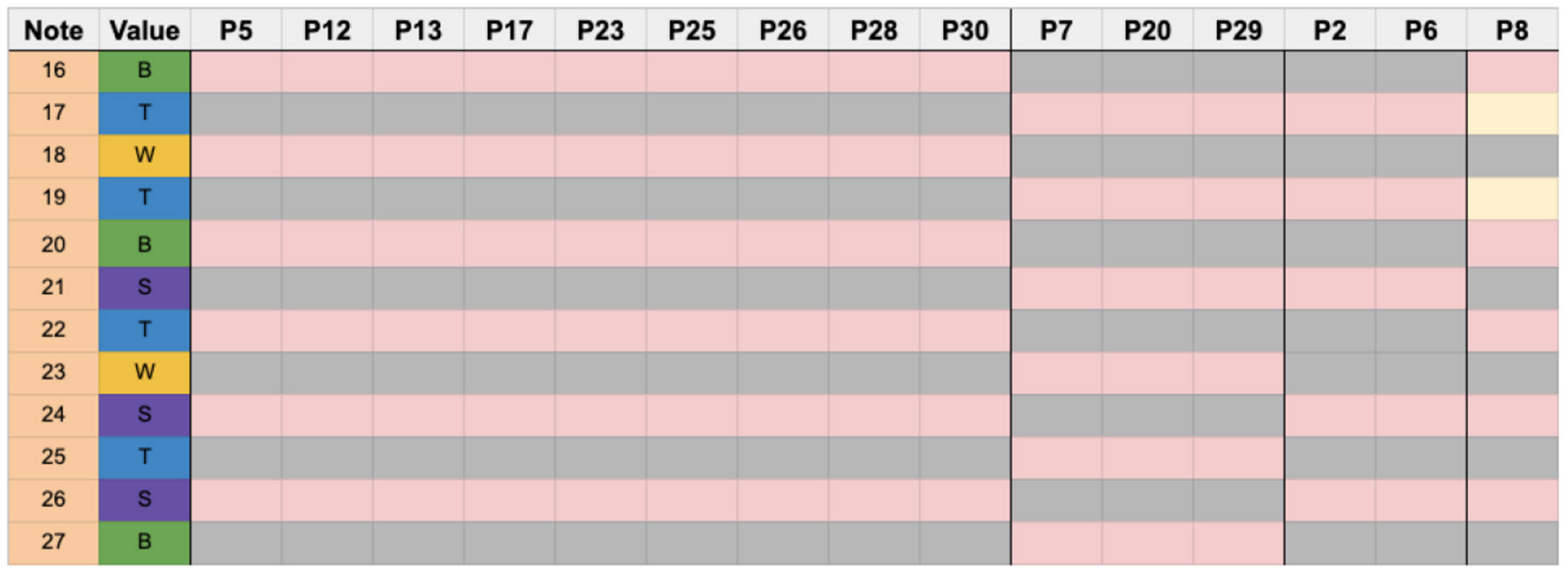
Participants who used a strategy other than that of dominant hand lead.

The largest number of participants in this region chose a dominant hand lead strategy, seen in Figure 8, followed by an alternating strategy beginning with the dominant hand, seen in Figure 9, demonstrating the popularity of our two model sticking patterns.

It is interesting to note that the only deviations that occurred from the model sticking patterns in this example were either a reversal of dominant hand lead (P2 and P6) or, in the case of P8, where the only reason we categorized their strategy as being different was due to the errors that were made.

As shown in Figure 9, starting on the right with P5 and up to P30, the participants exhibited the alternating model sticking strategy. For P7, P20, and P29 we see an alternating sticking pattern as well but in reverse starting with the non-dominant hand. Most notably, P2 and P6 used a strategy completely opposed to the dominant hand lead: non-dominant hand lead. P8 had two ghost notes, but if not for those seems to have employed an alternating sticking pattern beginning with the dominant hand.

### 5.6. Stable rhythm 2–measure 20

**Figure 10:**
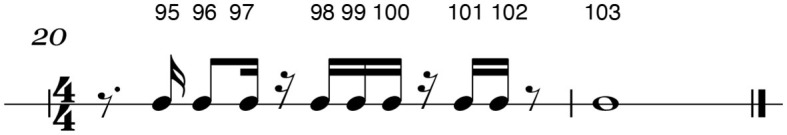
Measure 20 with note sequence identification.

Measure 20, seen in Figure 10, contains more syncopation than measure 6, but only in the first beat. All strokes for this measure fit within a sixteenth note grid, making this a stable rhythm, albeit slightly more complex than measure 6.

Due to the error threshold in this section, any participants who made more than one error, including a ghosted note, were not included in the analysis. For measure 20, participants P17, P19, P23, and P30 were excluded, leaving a total of 27 participants.

**Figure 11:**
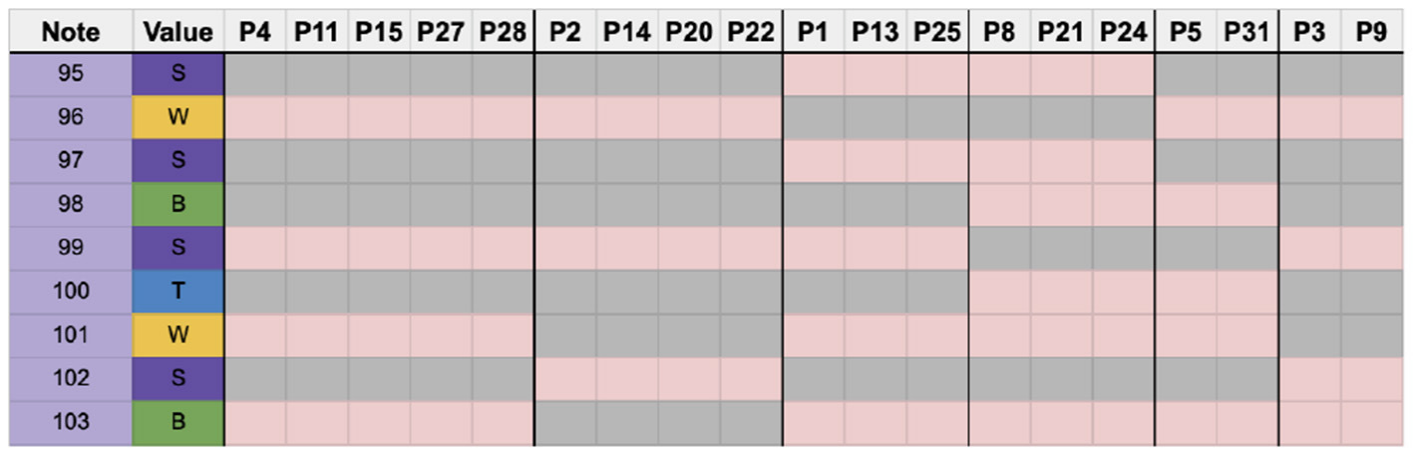
Chart showing the highest number of shared sticking choices from highest to lowest.

Overall the sticking strategies were more varied than in measure 6. Interestingly, only 2 participants (P5 & P31) chose to use a dominant hand lead sticking strategy. Five participants chose a sticking pattern that began as dominant hand lead but quickly switched to alternating seen in Figure 11 (P4, P11, P15, P27, P28). Only 3 participants used an alternating strategy (P1, P13, P25), meaning the other five groups of participants here chose identical strategies that did not resemble either dominant hand lead or alternating (Gworek, 2017).

**Figure 12:**
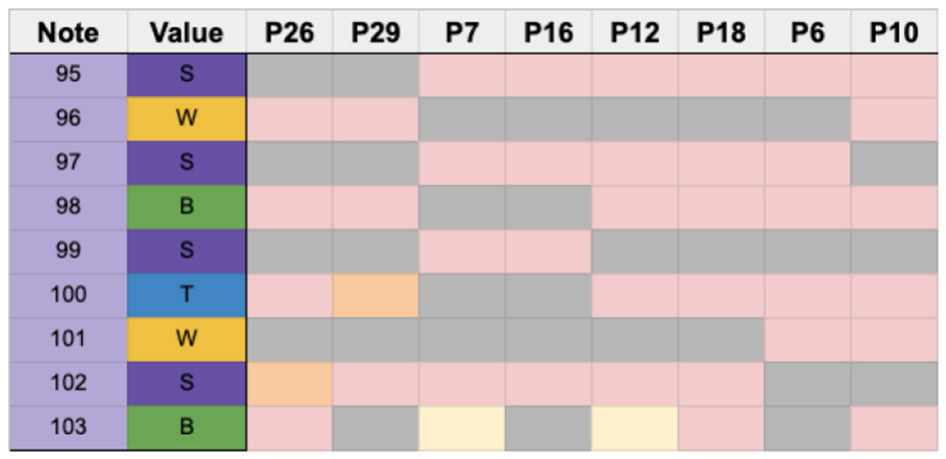
Chart showing the participants with no common sticking choice.

Finally, eight participants chose unique sticking strategies for this measure, as seen in Figure 12.

Overall, 14 different sticking strategies were identified among the participants, a large increase compared to measure 6.

### 5.7. Unstable rhythm 1–measure 11

**Figure 13:**
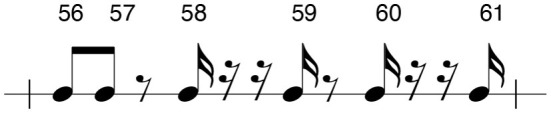
Complex syncopation in measure 11 with note sequence identification.

Measure 11, shown in Figure 13, was defined as unstable due to the prevalence of syncopated sixteenth notes and groups of sixteenth note rests.

The error threshold for this region was two, and only P30 was excluded in this analysis, leaving a total of 30 participants to analyze in this region.

**Figure 14:**
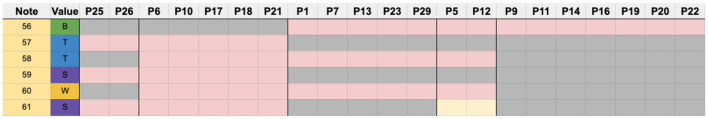
Chart showing participants who used identical strategies in measure 11 grouped together.

Figure 14 shows participants who used identical strategies in this measure. Seven participants chose the same sticking strategy, using their dominant hand to begin the section and the rest of the notes were played with the non-dominant hand (P9, P11, P14, P16, P19, P20, and P22).

In another case, five participants employed an alternating strategy which started with the dominant hand (P1, P7, P13, P23, and P29). Interestingly, five other participants (P6, P10, P17, P18, and P21) chose to do the inverse sticking of the dominant-lead group, thus leading with the non-dominant hand and completing the rest of the section with the dominant hand. Two participants (P5 and P12) chose an alternating strategy that started with the dominant hand but ended with a ghosted note. In contrast, two others chose an alternating strategy that began with the non-dominant hand (P25 and P26).

**Figure 15:**
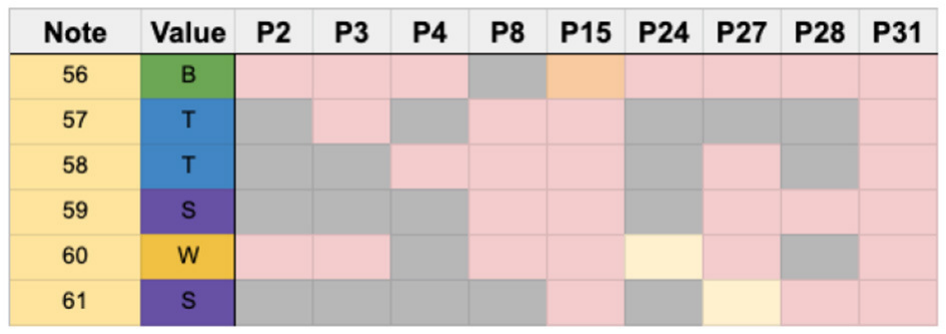
Chart showing the different strategies used in measure 11. P30 is removed due to the high number of errors.

Nine out of 30 participants used unique strategies that were not replicated by any other player (Figure 15).

Compared with measure 6, which contained stable rhythms, the sticking strategies in measure 11 were more varied. For example, the largest number of participants who used an identical sticking strategy was seven.

Overall, 14 different strategies were identified among the participants in this region.

### 5.8. Unstable rhythm 2–measure 13

**Figure 16:**
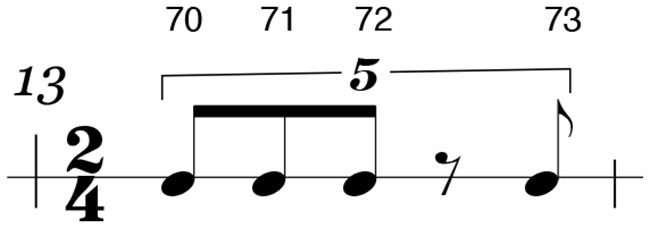
An irregular tuplet in measure 13.

**Figure 17:**
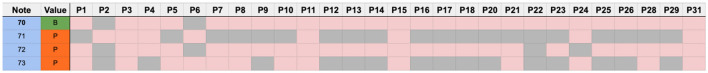
Chart showing the sticking choices of participants who successfully performed measure 13.

Measure 13, shown in Figure 16, has a small number of notes; therefore, the error threshold was set to one. P30, P19, and P27 were not considered in these results. The 28 performances of participants who successfully played measure 13 are shown in Figure 17.

**Figure 18:**
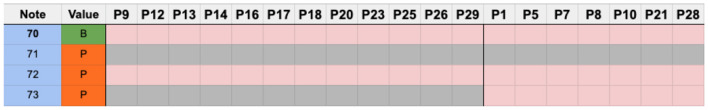
Chart showing participants who shared common sticking strategies clustered together.

**Figure 19:**
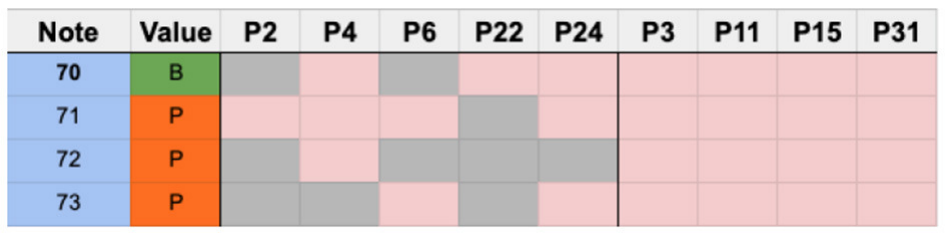
Chart showing participants who used unique strategies and a cluster that used only the dominant hand.

Out of 28 participants, 12 chose an alternating sticking strategy beginning with the dominant hand, while 7 participants chose a different sticking strategy which resembled a paradiddle beginning with the dominant hand. A paradiddle is a rudiment which begins with an alternation and then a double, such as RLRR or LRLL. Four participants used their dominant hand for all the notes, and the remaining 5 chose a variety of unique strategies. Figure 18 shows participants who shared common sticking choices. Figure 19 shows participants who chose unique strategies.

Overall, we noticed a clear trend toward a common sticking strategy of alternating with dominant hand initiation. A total of eight different strategies were utilized among participants for this measure.

### 5.9. Percussion sticking data

All of the sticking data available from the study discussed in this paper, a collection of sight-reading sticking data, along with the annotated score, has been made publicly available for further analysis here: https://github.com/mmwanderley2/PercussionStickingData.

#### 5.9.1. Database organization

The structure of the sticking database takes the form of an organized spreadsheet organized primarily by player for each column and by individual notes in the score by row. Every individual performed note in the score corresponds to one of four possible outcomes and is represented in the database as follows; dominant hand (D), non-dominant hand (ND), ghost note (G), and error (E). Basic participant experience data and stated handedness can also be seen. In addition, each subsection of the score is outlined, including the individual note sequence and rhythmic function. The abbreviations for rhythmic functions can be seen in Figure 7.

## 6. Discussion

The principles of percussion pedagogy are generally structured to accommodate a performer's physical limitations, which includes recognizing the reliance on the dominant hand for sound quality and rhythmic accuracy (Peters, 1986; McClaren, 1994). Sticking strategies in traditional pedagogy align with this preference, emphasizing the dominant hand for rhythmically important notes. Our results confirm the influence of these pedagogical principles, as performers strongly favored their dominant hand for strong beats, partially supporting our hypothesis, and consistent with prior research (Bacon et al., 2014).

However, it is noteworthy that a few participants did not show a clear preference for the dominant hand but still performed well, suggesting that model sticking strategies for teaching may not correspond directly with better performance skills in sight-reading. For example, Participants P2 and P6 deviated from the dominant hand lead strategy, starting sections with the non-dominant hand and employing alternative sticking choices. Despite these atypical patterns, they exhibited an error free performance in the sight-reading task, suggesting that strict adherence to model sticking patterns may not necessarily improve performance skills.

The emergence of alternative and hybrid sticking strategies, deviating from the model patterns, was observed when participants did not strictly follow the prescribed sticking patterns. These findings neither fully confirm nor refute our first hypothesis, indicating that other sticking patterns or hybrid approaches are also possible. However, our first hypothesis is supported by the prevalence of the two model sticking patterns in measure 6.

While increased rhythmic complexity did lead to a wider variety of sticking strategies, it was not solely responsible for this phenomenon. In measure 20, labeled as rhythmically stable, we observed the same number of different sticking strategies as in measure 11, which was considered unstable. The combination of idiosyncratic notation and rhythmic complexity may contribute to this effect. Additionally, handedness played a role in sticking choice, with left-handed players exhibiting a wider variety of sticking patterns even in stable and conventional regions of the score. This trend aligns with studies suggesting that left-handed individuals often possess more ambidextrous abilities (Lombana et al., 2022).

### 6.1. Use of model stickings

The results of our study showed a preference for the dominant hand lead model sticking pattern among the participants for the entire sight-reading score as well as the selected measures of analysis. This was also consistent across both stable and unstable measure examples (mm. 6, 11, 13, and 20) seen in Table 4. Hamming distances further confirmed this observation.

Musical instrument training involves demonstrations by teachers, particularly in percussion training where sticking instructions for specific musical passages are common. To consider how an instructor's personal preferences may influence students is highly informative. If a teacher favors the right-hand lead strategy, this bias may be passed on to the student. Similarly, if a teacher emphasizes perfect symmetry, students are likely to be trained in alternate sticking and may prefer it. Evaluating student-teacher learning styles can provide further insights, as the prevalent “maestro” pedagogical method and power dynamics strongly influence student behavior (Zhukov, 2007). For instance, percussionists with military-style training, such as those in marching bands, are taught specific sticking strategies with varying difficulty levels and visual style considerations. Studying sticking choices in this context offers valuable insight into the influence of pedagogical style and teacher-student relationships.

### 6.2. Role of stable rhythms

We chose stable rhythms that could clearly be subdivided or placed easily into a sixteenth-note grid, with little to no syncopation. Our hypothesis that model sticking would be prevalent in stable rhythms was confirmed by the fact that most participants utilized model sticking patterns in the first stable rhythm (measure 6). Not only was this an easy rhythm to execute, but it was also familiar. Many, if not all, of the percussionists would have encountered this same rhythmic pattern many times before in training and performance. For this reason and due to its simplicity, participants tended to rely more on model sticking patterns, and strategies were largely homogeneous among participants.

However, the second stable rhythm (measure 20) yielded a much wider variety of patterns. The average hamming distance between the two model stickings were only 0.11 strokes apart across all participants in this measure. The paired Wilcoxon Rank testing, seen in Table 4, further demonstrated the lack of significance in the observed difference between the two model stickings. Though there were two groups of three performers each who chose dominant hand lead and alternating, many other strategies were employed. Fourteen sticking strategies were used in the second stable rhythm, whereas only five were used in the first.

There are two likely reasons for this. First, measure 20 is less rhythmically stable than measure 6. Most of the measure contains no syncopation, though it begins with a syncopated rhythm. Secondly, the rhythm is not notated in a simple manner. Since there is always more than one way to notate a rhythm, some forms of notation can be considered to be more straightforward than others. In this measure, there are more sixteenth-note rests than necessary to communicate the rhythm (when considering just note-onset times) potentially making it more difficult for the participant to parse the notation.

### 6.3. Role of unstable rhythms

As shown in the examples in measures 11 and 13, unstable rhythms impact a performer's sticking strategy. Both measures yielded a wider variety of sticking patterns than in the stable rhythms, which confirms our hypothesis.

In measure 11, this rhythm was defined as unstable because of the unidiomatic notation and the amount of syncopation present, even though all the notes could be placed easily into a sixteenth-note grid. One group of participants (seven performers) utilized the alternating strategy, but no participant used a dominant hand lead strategy. In measure 13, where participants encountered a quintuplet, we found only eight strategies employed. Still, it is important to note that only one was a model sticking strategy. For example, 12 participants used an alternating strategy, but no one used a dominant hand lead strategy, which is similar to what was found in measure 11.

This can be explained by the fact that dominant hand lead is impossible with a quintuplet rhythm spanning an entire bar of two beats. Since it does not fit in a standard rhythmic grid of sixteenth notes but rather creates rhythmic tension with that grid, automation of the non-dominant hand while the dominant hand maintains a steady beat is rendered useless as a potential strategy.

### 6.4. The role of notation in stable and unstable rhythms

The sight-reading score used in this experiment serves as a form of musical instruction, where each note represents a specific action for the performer. The clarity of visual presentation largely impacts our interpretation of the intended expression, aligning with previous research on note spacing in sight-reading to enhance legibility and performance (Stenberg and Cross, 2019). Parsing the unidiomatic notation found in our sight-reading score may share similarities with target-distractor research, where unconventional notation can complicate the reading process for performers (Chang and Gauthier, 2020).

**Figure 20:**
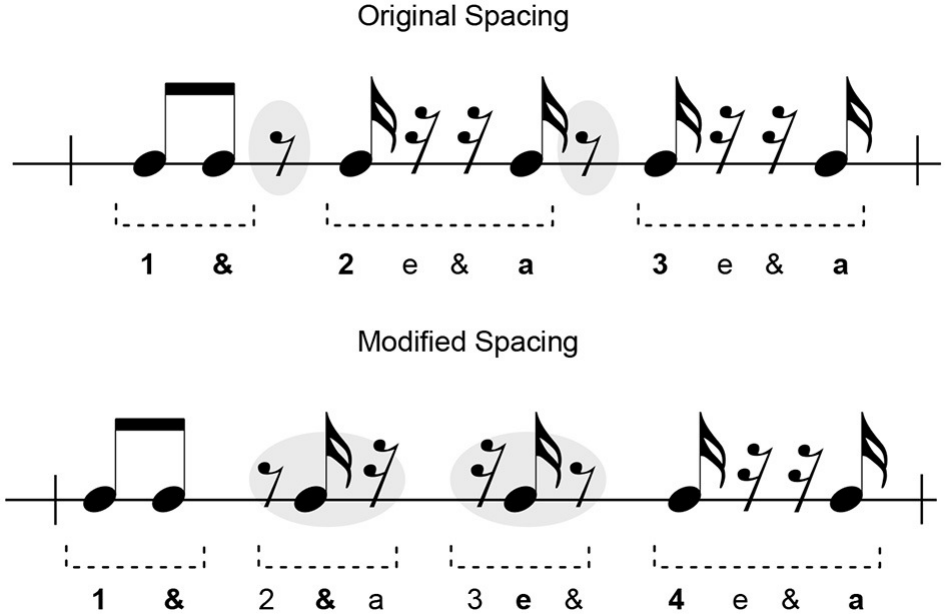
Measure 11 as shown in its original with annotation suggesting possible perceived note groupings and counting scheme (playable counts are emboldened). The modified spacing below is designed to preserve identification of major beat markers, as evidenced in the counting scheme. The gray highlights indicate the location of the loose eighth-note rests in the original presentation and location in the re-notation.

These findings suggest that visual perception and the “busyness” of the image may also influence performers (Rosenholtz et al., 2007). Notation examples with unidiomatic characteristics, such as cluttered note/rest groupings, could have a unique impact on performers' sticking choices, regardless of rhythm stability. Measure 11 exemplifies the influence of information presentation factors. The observed sticking patterns in this measure showed greater deviations from the model stickings compared to the entire score (as seen in Tables 3, 4). We suspect this is partly due to the unidiomatic notation. To illustrate, Figure 20 demonstrates how the original presentation of measure 11 could potentially confuse sight-readers. The spacing of note groups could create the illusion of a 3/4 measure due to the groupings of sixteenth-notes and adjacent rests. The placement of two eighth-note rests (highlighted in gray) lacks a clear association with the strong and weak beat markers. In contrast, the modified version, following (Stenberg and Cross, 2019) approach, preserves the major beat markers in relation to note groupings. The highlighted clusters indicate the correct placement of the aforementioned eighth-note rests. Although this requires validation in future tests, we predict that the second notation would be easier to read due to the preservation of strong-beat and weak-beat groupings.

### 6.5. Impact of handedness

While primarily focusing on hand dominance, our study revealed intriguing patterns associated with left- and right-handedness, meriting further investigation. Regarding model stickings, both left- and right-handed players deviated similarly from the alternate sticking strategy, with percentages of 53.20 and 56.12% respectively. However, right-handed performers showed a stronger resemblance to the dominant hand lead sticking strategy, with only a 32.11% difference, compared to left-handed individuals who exhibited a 58.06% difference. These percentages represent the hamming distances as a percentage of total playable notes in the sight-reading score. These findings align with observations suggesting that left-handed individuals tend to demonstrate more ambidextrous behavior than their right-handed counterparts (Gutwinski et al., 2011).

The use of sticking strategies other than the model ones by left-handed players suggests a potential influence of handedness on sticking choices. For instance, in measure 6, characterized by stable rhythm and conventional notation, only one out of 16 left-handed players employed a dominant hand lead strategy. Similarly, among nine participants, only one left-handed performer used the alternating strategy. Among the remaining six players who employed other strategies, half were left-handed.

This discrepancy between right and left-handed players may be attributed to a right-hand bias in percussion pedagogy, where teachers often teach a “right-hand lead” approach even to left-handed students. Such bias could contribute to the slightly greater diversity in stickings observed among left-handed players, as left-handed individuals have been shown to exhibit more symmetry than their right-handed counterparts.

### 6.6. Study limitations

Our study focused on the performance behaviors of participants on a single timpano, within the context of timpani performance. Scores and compositions often involve multiple timpani, requiring complex sticking cross-over techniques when transitioning between drums to maintain preferred sticking patterns and smooth sound. Therefore, our findings specifically pertain to single-drum performance and serve as a foundation for future research. Subsequent investigations should explore the impact of more intricate playing techniques (e.g., rolls, accents, and muting) across multiple drums in relation to handedness and sight-reading. These factors make our findings relevant to broader percussion performance practice and bi-manual interaction. We also acknowledge that the preferred setup style (German; low-high, or American; high-low) for a given performer may have an effect on their sticking pattern even in the context of playing a single timpano. Future studies on percussion performance and sticking choices can draw stronger inferences regarding handedness by incorporating independent evaluations of participants' handedness, as well as timpani setup preferences.

In terms of note timing and error detection, our study relied on the percussion expertise of co-authors Bacon, Jackson, and Marandola. While their training ensured accurate evaluation of percussion performance, employing computational methods to detect precise tempo drift after switching off the metronome could enhance future studies. Furthermore, analyzing additional performance error trends related to time-signature changes and polyrhythmic tuplets may yield valuable insights.

Lastly, muting behavior was observed in 7 out of 31 performers. Muting performers, who used their non-performing hand to influence note duration, exhibited similar performance behaviors to the rest of the group in key factors, except for a higher overall error rate: 9.01% for muting players compared to 2.22% for non-muting players. This disparity in error rates may be attributed to divided attention between note onset and duration, affecting both the playing and muting hands, warranting further investigation.

## 7. Conclusion

In this paper, we studied how musical context can affect sticking choice with percussionists in a sight-reading exercise. More specifically, we chose two model sticking patterns in pedagogical literature that we hypothesized would emerge more often when the participants encountered more stable rhythms.

We proposed a methodology which included a score designed for this experiment, with regions notated explicitly to test the effect of rhythmic complexity on sticking choice. A total of 31 participants, all highly trained percussionists, took part in the experiment. Sticking data obtained from the performances was compared to model sticking patterns. We used an error classification system and threshold for handling participants that made mistakes in specific regions. In addition, a system of beat classification was used to analyze rhythmic complexity and the function of model sticking patterns.

Sticking patterns reflect personal choices or strategies, as no two performers employed exactly the same sticking pattern in our study. However, it is possible to find preferences and patterns shared by many; percussionists strongly preferred to use their dominant hand for strong beats and start the beginning of phrases. Moreover, many players often used the two model sticking strategies (dominant hand lead and alternating), especially in sections that were based on stable rhythms, including those with idiomatic notation. When model sticking patterns were not strictly adhered to, other strategies emerged as a hybrid between the two models or some original pattern. Furthermore, sticking patterns varied more in sections that were either less rhythmically stable or where the notation was obfuscating the structure of the rhythm. A paired Wilcoxon Rank test of the results further demonstrated that for the entire score, the preference toward the dominant hand lead model was significant.

Although it has been proposed in the literature that performances improved when using one of two model sticking strategies, dominant hand lead or alternating, we found out that several participants used a strategy opposite to dominant hand lead or a hybrid approach, and this did not seem to affect their performance abilities. This could be due to experience, but it suggests that the model sticking strategies so commonly used in method books and teaching might not directly correlate with improved performance in sight-reading tasks. Once more, the overall performance conditions and expectations of sight-reading and a rehearsed musical performance may require different sticking strategies, challenging the “one size fits all” approach commonly seen in percussion pedagogical literature. Continued research into how sticking patterns evolve as players become more familiar with a given score may shed light on this topic. More studies on percussion sticking strategies are needed to validate this finding, as it carries important implications for pedagogical practice.

The proposed methodology could be used to test future hypotheses related to sticking choice. For example, the effect of handedness could be further explored if more left-handed players are recruited. The methodology could be used to understand the impact of notation on sight-reading performance. In addition, we found that notation may also affect sticking behavior, as note spacing, visual presentation, and unidiomatic notation could influence a player's performance behavior. Lastly, we have noted a trend among left-handed players of using unique strategies even when playing stable rhythms with efficient notation, suggesting that handedness may also play a role in sticking choice.

## Data availability statement

The raw data supporting the conclusions of this article will be made available by the authors, without undue reservation.

## Ethics statement

The studies involving human participants were reviewed and approved by REB-II McGill Ethics Committee. The patients/participants provided their written informed consent to participate in this study.

## Author contributions

The original impetus for this research originated in discussions between MW and FM about movement variability in motion capture data of timpani performances. BB devised the experimental methodology in collaboration with FM and MW, composed the original score, and captured the performances of the first 4 subjects. IM took over and expanded BB's results, capturing the performances of 10 more performers. SJ captured the last set of timpanists and performed preliminary analysis on the complete data set. MW and FM have overseen this research over the past nine years. All authors contributed to the article and approved the submitted version.
